# From awareness to action: determinants of zoonotic risk perception and protective practices among farm and livestock workers

**DOI:** 10.3389/fpubh.2026.1818149

**Published:** 2026-06-16

**Authors:** Yong Shen Ooi, Norefrina Shafinaz Md Nor, Nur Azalina Suzianti Feisal, Wai Yan Cheah

**Affiliations:** 1 Centre of Research in Development, Social and Environment (SEEDS), Faculty of Social Sciences and Humanities, Universiti Kebangsaan Malaysia, UKM Bangi, Selangor Darul Ehsan, Malaysia; 2 Environmental Management Programme, Faculty of Social Sciences and Humanities, Universiti Kebangsaan Malaysia, UKM Bangi, Selangor Darul Ehsan, Malaysia; 3 Universiti Teknologi MARA (UiTM) Penang Branch, Bertam Campus, Kepala Batas, Pulau Pinang, Malaysia; 4 Department of Biological Sciences and Biotechnology, Faculty of Science and Technology, Universiti Kebangsaan Malaysia, UKM Bangi, Selangor, Malaysia; 5 MSU Center for Climate Resilience and Strategy (m-CREST), Management and Science University (MSU), University Drive, Off Persiaran Olahraga Section 13, Shah Alam, Selangor, Malaysia; 6 Department of Diagnostic and Allied Health Science, Faculty of Health and Life Sciences, Management and Science University (MSU), University Drive, Off Persiaran Olahraga Section 13, Shah Alam, Selangor, Malaysia

**Keywords:** agricultural workers, environmental management, occupational exposure, one health, risk perception, zoonotic diseases

## Abstract

**Introduction:**

Zoonotic disease prevention among farm and livestock workers depends not only on awareness, but also on how workers perceive occupational risk and the feasibility of protective action. This systematic literature review examined how farm and livestock workers perceive zoonotic disease risks and how these perceptions relate to everyday practices and working environments.

**Methods:**

The review followed PRISMA guidance and used a PICO-based question formulation. Searches were conducted in Web of Science and Scopus. A total of 28 studies were included, covering dairy, pig, poultry, and mixed livestock systems across Africa, Asia, Europe, and the Americas. Evidence was synthesized thematically, with attention to risk perception, knowledge gaps, protective practices, and socio-environmental determinants.

**Results:**

Workers generally recognized that animals can transmit disease to humans, but their understanding of specific pathogens, transmission routes, high-risk tasks, and preventive measures was uneven. Knowledge gaps were most evident for endemic, environmentally mediated, and parasitic infections. A persistent perception-behavior gap was observed, particularly where personal protective equipment, sanitation, clean water, supervision, and institutional support were limited.

**Discussion:**

Protective practices were shaped by socio-cultural norms, gendered labor roles, livelihood pressures, environmental conditions, workplace safety climate, and employer or institutional support. The findings highlight the need for multi-level One Health strategies that combine worker-centered education with improvements in infrastructure, regulation, organizational safety culture, and cross-sector collaboration. The review also identifies the need for future research in under-studied agricultural settings, including plantation contexts.

## 1 Introduction

Zoonotic diseases are infections that are naturally transmitted between animals and human population, arising through direct contact, indirect contact via contaminated environments, vector bites, food and water, or inhalation of infectious material. This animal-human-environment interface is where many everyday tasks of farm and plantation work take place, which is why primary producers, farm households, and agricultural workers are repeatedly exposed during animal handling, calving and farrowing, milking, slaughter and carcass processing, manure management, and contact with fomites in animal housing. The World Health Organization (WHO) and partner agencies frame this challenge through One Health, a collaborative approach that links human, animal, and environmental health to prevent and control threats that move across species and systems. Evidence from agricultural settings underscores that farms and dairies are working environments with a high-risk of exposure to pathogens such as Salmonella, pathogenic Escherichia coli, Campylobacter, and Cryptosporidium, with transmission occurring through ingestion, skin contact, aerosols, and contaminated clothing and surfaces (1, 2).

Plantation and farm settings concentrate human animal environment contact through livestock husbandry, wildlife proximity, vector exposure, and routine use of natural water sources. Environmental and occupational drivers such as contact with flood water and mud and work in wet cultivation are repeatedly implicated in leptospirosis risk, illustrating how landscape, climate and daily tasks intersect to shape exposure (3). In tropical forest and forest edge economies, zoonotic malaria adds further complexity as contact with macaques and Anopheles mosquitoes is affected by deforestation and land use change (4). These realities align with a One Health perspective that integrates human, animal and environmental health to prevent, detect and respond to zoonotic threats in a coordinated way (5).

Workers' willingness to adopt safety precautions is closely shaped by their perception of risk. Risk perception involves workers' judgments about the likelihood and severity of harm, as well as the perceived value, feasibility, and cost of protective action. Risk perceptions among workers are shaped by an accumulation of knowledge, emotional responses, workplace climate and peer culture and also affected by resource constraints (6, 7). In a case from dairy farms, for instance, employees working under supportive supervision perceived their work environment more favorably, had less occupational exposure and felt less threatened from zoonoses because safe practices and normative behaviors toward those behaviors among their coworkers and the farmers were observed (2). If such contextual factors persist throughout an entire occupation where workers interact repeatedly with animals or contaminate their surroundings, such workers are not consistently inclined to adopt such protective practices in their daily routines and tasks even when they are fully informed. Therefore, this systematic review synthesizes evidence on how zoonotic risk perception among farm and livestock workers relates to occupational exposure, protective behavior, and working environments.

## 2 Research methodology

### 2.1 Reporting framework and review protocol

PRISMA, or Preferred Reporting Items for Systematic Reviews and Meta-Analyses, is a widely recognized guideline for conducting systematic literature reviews. Publication standards generally aim to equip authors with essential information to evaluate a review's quality and rigor. PRISMA emphasizes transparent reporting, especially for reviews involving randomized trials, and provides a valuable framework for systematic reviews across diverse research domains (8). Previous studies have also outlined systematic review methodologies specifically for veterinary and agricultural contexts, applicable to zoonotic diseases (9).

### 2.2 Formulation of research question

The research question for this study was formulated using the PICO framework, designed to help researchers construct structured, focused questions for systematic reviews (10, 11). PICO consists of four key components: Population or Problem, Intervention or Interest, Comparison or Context, and Outcome. This study incorporates three main elements: agricultural workers, primarily in livestock and mixed farming systems, as the population, risk perception of zoonotic diseases as the interest, and working environments as the context. Thus, the study addresses these questions: (i) What are the key knowledge gaps and behaviors influencing zoonotic disease risks among workers? (ii) How do socio-cultural, economic, and environmental factors shape risk perception? (iii) Which policies and interventions effectively reduce zoonotic disease risks, or are currently needed?

### 2.3 Systematic searching strategies

**Figure 1:**
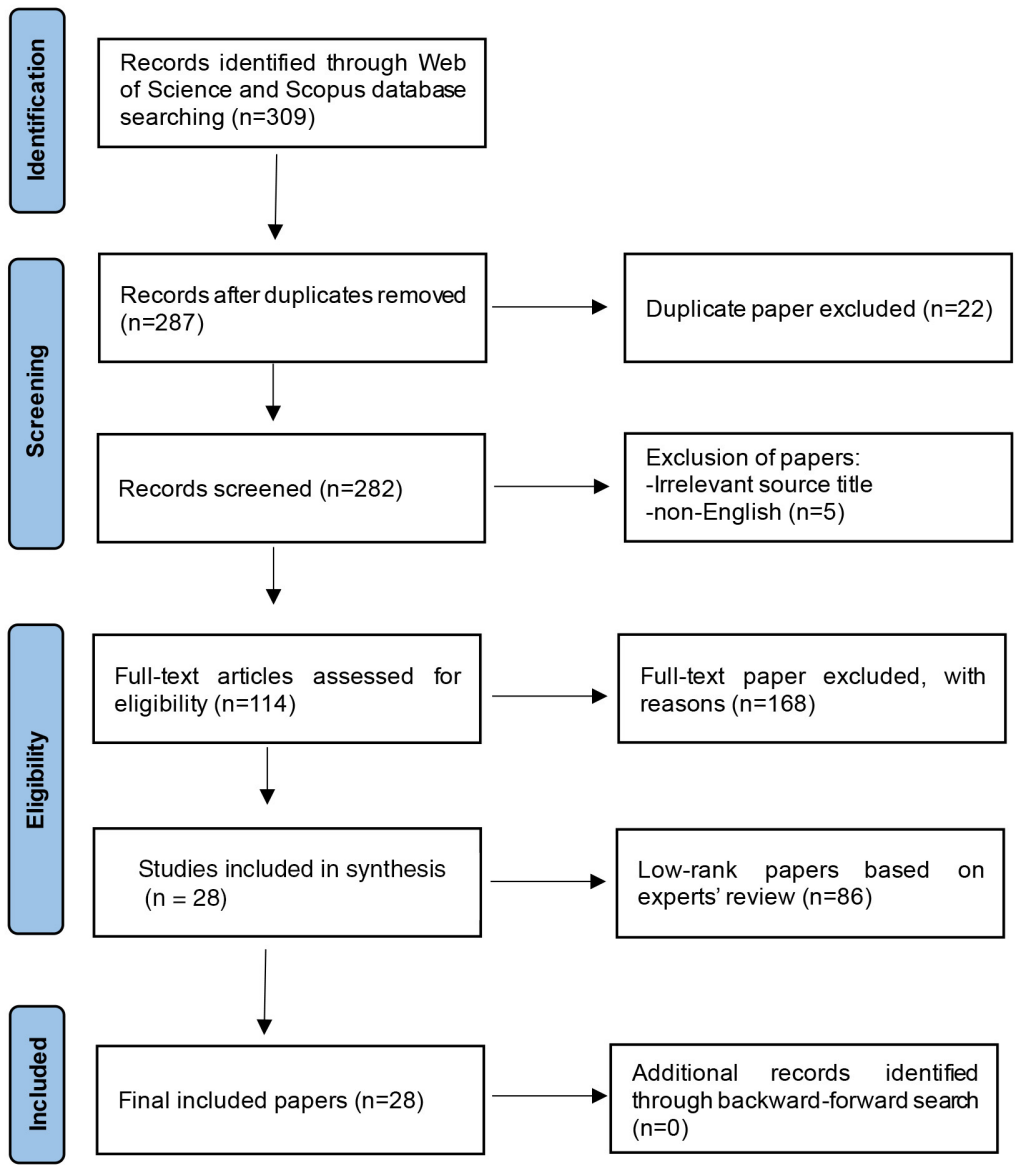
Flow diagram of the systematic literature review (SLR) process.

The search strategy comprised three sequential phases that are visualized in Figure 1. These phases were identification, screening, and eligibility, culminating in inclusion.

#### 2.3.1 Identification

Identification refers to the process of selecting relevant keywords based on the research questions. In this study, three primary keywords were used: zoonotic disease, risk perception, and plantation workers. These terms were expanded with synonyms, related concepts, and variants derived from a structured research question framework (12). The process involved consulting online vocabularies, reviewing terms from earlier studies, incorporating suggestions from the Web of Science and Scopus databases, and drawing on expert input.

Study refined a series of keyword strings to search for relevant publications in the Web of Science and Scopus shown in Table 1. These databases were selected because they provide broad access to academic literature and detailed citation information. They were also preferred for their rigorous peer-review standards, which ensure the quality of indexed articles and uphold the impact factor rankings of journals (13, 14). The search strategy across both databases led to the identification of 309 articles that met the study's research objectives.

**Table 1:** Article search strings.

No.	Database	Search strings
1	Web of Science	**TOPIC**: (zoonotic OR zoonoses OR animal-borne disease) AND **TOPIC**: (plantation OR agriculture OR farm OR crop OR estate) **Refined by: TOPIC** (worker OR staff OR employee) AND **TOPIC**: (risk perception OR knowledge OR attitude OR practice OR awareness OR judgement OR belief OR understanding)
2	Scopus	TITLE-ABS-KEY (zoonotic OR zoonoses OR animal-borne disease) AND ((plantation OR agriculture OR farm OR crop OR estate) AND (worker OR staff OR employee)) AND (risk perception OR knowledge OR attitude OR practice OR awareness OR judgement OR belief OR understanding)

#### 2.3.2 Screening

Duplicate removal excluded 22 records, leaving 287 unique items. Title and abstract screening then limited the corpus to peer reviewed empirical studies in English, which removed a further 5 non-English records and advanced 282 items to eligibility assessment.

#### 2.3.3 Eligibility

Eligibility decisions were based on detailed reading of titles and abstracts against predefined criteria. Exclusions at this stage covered studies on zoonoses outside agricultural or plantation settings, populations other than plantation or farm workers, non-empirical items such as reviews and opinion pieces, and book chapters. After applying these criteria 114 articles remained for full systematic review.

#### 2.3.4 Quality appraisal and risk of bias assessment

Methodological quality of the included studies was appraised using the Mixed Methods Appraisal Tool (MMAT) (15). Because the review included quantitative, qualitative, and mixed-methods studies, each included article was first classified by study design and then assessed using the corresponding MMAT criteria. Quantitative descriptive studies were evaluated against five criteria covering sampling relevance, sample representativeness, appropriateness of measurements, risk of non-response bias, and appropriateness of statistical analysis. The qualitative study was appraised using the MMAT qualitative criteria, and mixed-methods studies were assessed at the component level, including the qualitative component, the quantitative descriptive component, and the mixed-methods integration component. Ratings were recorded as “Yes,” “No,” or “Can't tell.” In line with MMAT guidance, no overall numeric score was calculated. Detailed study-level appraisal results are presented in Supplementary Tables S1–S3. The review included 28 studies comprising quantitative, qualitative, and mixed-methods evidence. Most quantitative descriptive studies met the criteria for relevance of sampling strategy and appropriateness of statistical analysis, whereas the most frequent limitations concerned sample representativeness and incomplete reporting of non-response bias. The single qualitative study met all five MMAT qualitative criteria. The mixed-methods studies generally showed a clear rationale for using mixed methods and reasonable integration of qualitative and quantitative components.

#### 2.3.5 Data analysis

An integrative review approach was used to synthesize quantitative, qualitative, and mixed methods evidence into a coherent analysis (Table 2). Thematic analysis guided coding and category development. In stage one, information aligned to the three research questions was extracted from each of the 28 studies. In stage two, a structured coding process organized the extracted material into themes and sub themes that capture recurring patterns and cross study connection. In addition to data aligned with the research questions, descriptive study characteristics were extracted, including publication year, country, WHO region, worker or occupational setting, study design, and type of zoonotic disease. These variables were used to summarize the spatial and temporal distribution of the included evidence before thematic synthesis.

**Table 2:** Background of the selected studies.

Research	Study country	Region (WHO)	Study methods	Key finding
Suolaniemi et al. (40)	Finland	Europe	QT	Knowledge moderate; risky habits common
Smith et al. (25)	Rwanda	Africa	QT	Radio dominant info source; gendered differences in familiarity
Rinchen et al. (38)	Bhutan	South-East Asia	QT	High awareness but low ‘adequate' knowledge; PPE seldom used
Rehman et al. (32)	Indonesia	South-East Asia	QT	TV and health workers key information channels
Rabinowitz et al. (39)	Romania	Europe	QT	High concern but low respiratory protection uptake
Pham-Thanh et al. (30)	Vietnam	Western Pacific	QT	Common farm routines did not reduce pig exposure
Palomares Velosa et al. (2)	United States	Americas	QT	Higher safety knowledge & positive climate lead to lower measured exposure
Nankam Chimi et al. (29)	Cameroon	Africa	QT	Low knowledge strongly tied to low-risk perception
Mahon et al. (1)	Ireland	Europe	QT	Veterinarians most-cited info source; well testing infrequent
Lowenstein et al. (41)	Ecuador	Americas	QL	Normalized risk; over the counter antibiotic access via feed shops
Kungu et al. (23)	Uganda	Africa	QT	Very low human-pig linkage understanding; poor sanitation; deworm pigs > people
Kulabako et al. (22)	Uganda	Africa	MX	Knowledge/practices variable; raw milk consumption reported
Kemal et al. (19)	Ethiopia	Africa	QT	Raw milk preference common; awareness limited
Hussain et al. (33)	Pakistan	Eastern Mediterranean	QT	Frequent tick exposure; PPE uncommon
Getahun et al. (18)	Ethiopia	Africa	QT	Awareness modest; raw milk consumption reported
Efrem et al. (20)	Eritrea	Africa	QT	Hired workers scored lower than family members
Dhakal et al. (31)	Nepal	South-East Asia	QT	JE exposure widespread; personal protection inconsistent; regional differences
Deneke et al. (17)	Ethiopia	Africa	QT	Consumption patterns sustain bovine TB risk
Deka et al. (34)	India	South-East Asia	QT	Glove use at abortion events extremely rare
Cao Ba et al. (35)	Vietnam	Western Pacific	MX	Belief antibiotics prevent infection; reluctance to report sick animals
Bissong et al. (28)	Cameroon	Africa	QT	Low KAP and low risk perception co-occur
Birhan (26)	Ethiopia	Africa	QT	Extensive systems & larger herds lead to higher burden
Ayim-Akonor et al. (27)	Ghana	Africa	QT	Biosecurity gaps; PPE uptake low
Ayebare et al. (24)	Uganda	Africa	MX	Near-zero repellent use; treated clothing uncommon
Atherstone et al. (21)	Uganda	Africa	QT	Inter-district movements; non-reporting of sick pigs; informal slaughter
Almaw et al. (16)	Ethiopia	Africa	QT	Genotypes consistent with endemic bovine TB; control gaps implied
Adenuga et al. (36)	Cambodia	Western Pacific	QT	Awareness that humans affected often low
Abukhattab et al. (37)	Palestine	Eastern Mediterranean	MX	Weak reporting; unsafe carcass disposal despite stated support

MX, Mixed Method; QT, Quantitative; QL, Qualitative.

## 3 Result

### 3.1 Study selection and quality appraisal

The database search identified 309 records. After removal of 22 duplicates, 287 unique records remained for title and abstract screening. Following eligibility assessment, 114 articles were retained for full-text review, of which 28 met the final inclusion criteria. Study selection is presented in Figure 1. Methodological quality was appraised using MMAT (2018). Most quantitative descriptive studies met the criteria for relevance of sampling strategy and appropriateness of statistical analysis, while the most frequent limitations concerned sample representativeness and incomplete reporting of non-response bias. The single qualitative study met all five qualitative MMAT criteria. Mixed-methods studies generally demonstrated a clear rationale for mixed-methods design and reasonable integration of components, although reporting of divergence handling and component-level quality was less consistent.

### 3.2 Characteristics of included studies: spatial distribution

**Figure 2:**
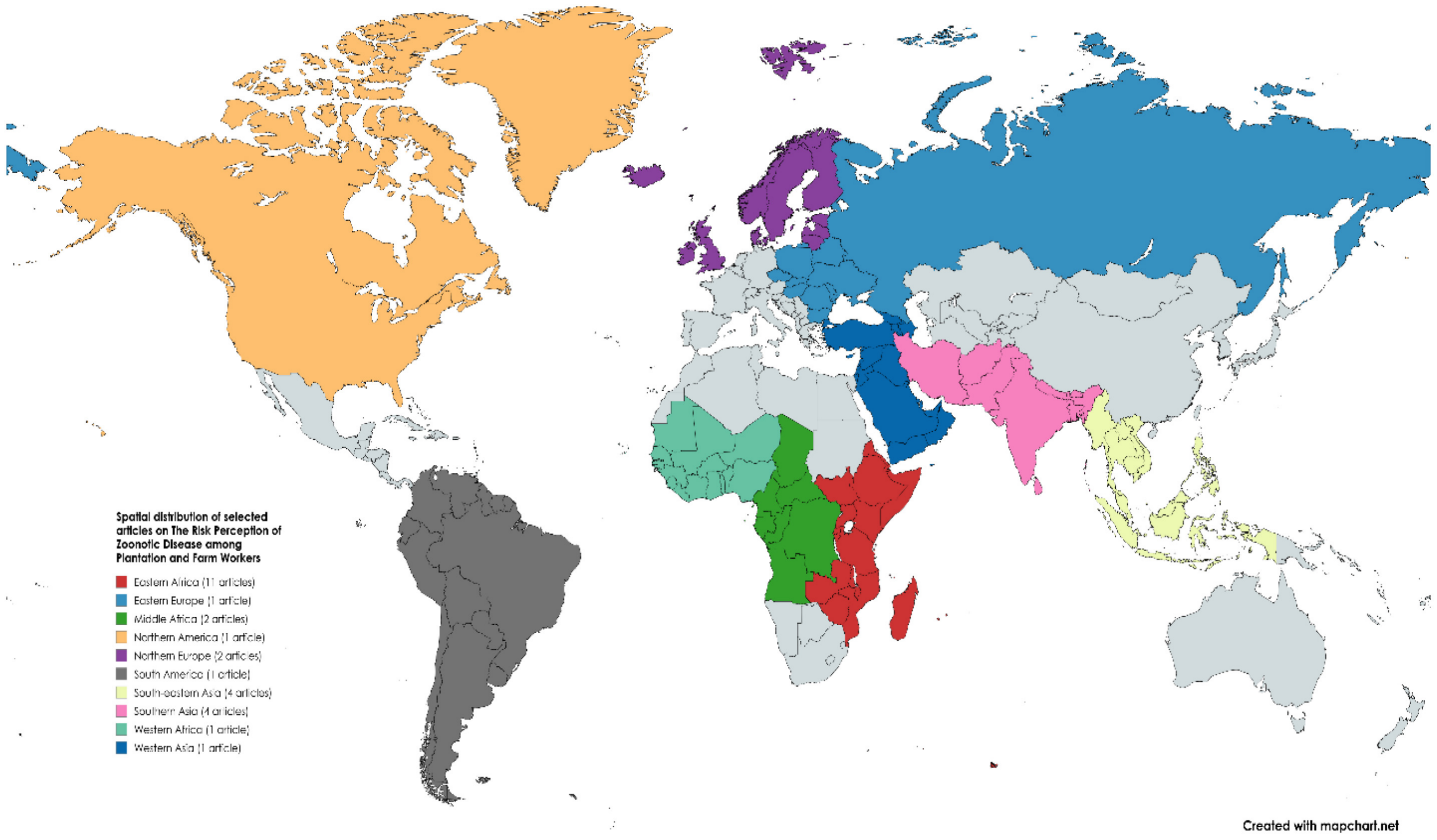
Spatial distribution based on the United Nation geoscheme of selected articles (Created using mapchart.net).

Spatial distribution (Figure 2) shows in in East Africa and the Horn, dairy and mixed livestock systems dominated the risk picture, with Ethiopia contributing repeated evidence on dairy-associated zoonotic risks, while Uganda and Rwanda highlighted value-chain exposure, tick-related risk, and gendered communication pathways. Together, these studies marked the region as a priority setting for dairy-focused and value-chain-aware prevention (16–26). In West and Central Africa, poultry and small ruminant systems show consistently low uptake of gloves and masks, weak carcass and abortion management, and low knowledge and low risk perception, indicating the need for task specific coaching and affordable supplies alongside messaging (27–29).

Meanwhile in South and Southeast Asia and the Middle East, there are mainly two ecologies dominate. First, vector and reservoir risks surround pigs and poultry in Vietnam, Nepal, Indonesia, and Pakistan, with very high pig flavivirus seroprevalence near Hanoi, widespread Japanese encephalitis exposure with inconsistent protection in Nepal, generally high knowledge among slaughterhouse workers in Indonesia, and common tick infestation with unsafe acaricide practices in Pakistan. Second, direct and food borne transmission risks appear in India and Vietnam and along pig value chains where awareness that humans are affected can be low. A broiler chain analysis from Palestine shows weak reporting and unsafe carcass disposal despite stated support for prevention. These studies point to ecology matched prevention and to closing intention and behavior gaps at the point of work (30–38).

Whereas in Europe and the Americas, Ireland shows uneven testing of private wells and continued raw milk use among dairy farmers with veterinarians as trusted advisors, Romania shows high concern but very low vaccination and respirator use among swine workers, and Finland shows half of dairy farmers with adequate cryptosporidiosis knowledge yet routine risky behaviors such as phone use in the cowhouse. Ecuador illustrates peri urban risk normalization and access to over-the-counter antimicrobials, while Colorado demonstrates that safety knowledge and positive supervisor and coworker perceptions are protective and that negative workplace climate is associated with higher measured exposure among dairy workers (1, 2, 39–41).

### 3.3 Characteristics of included studies: temporal distribution

**Figure 3:**
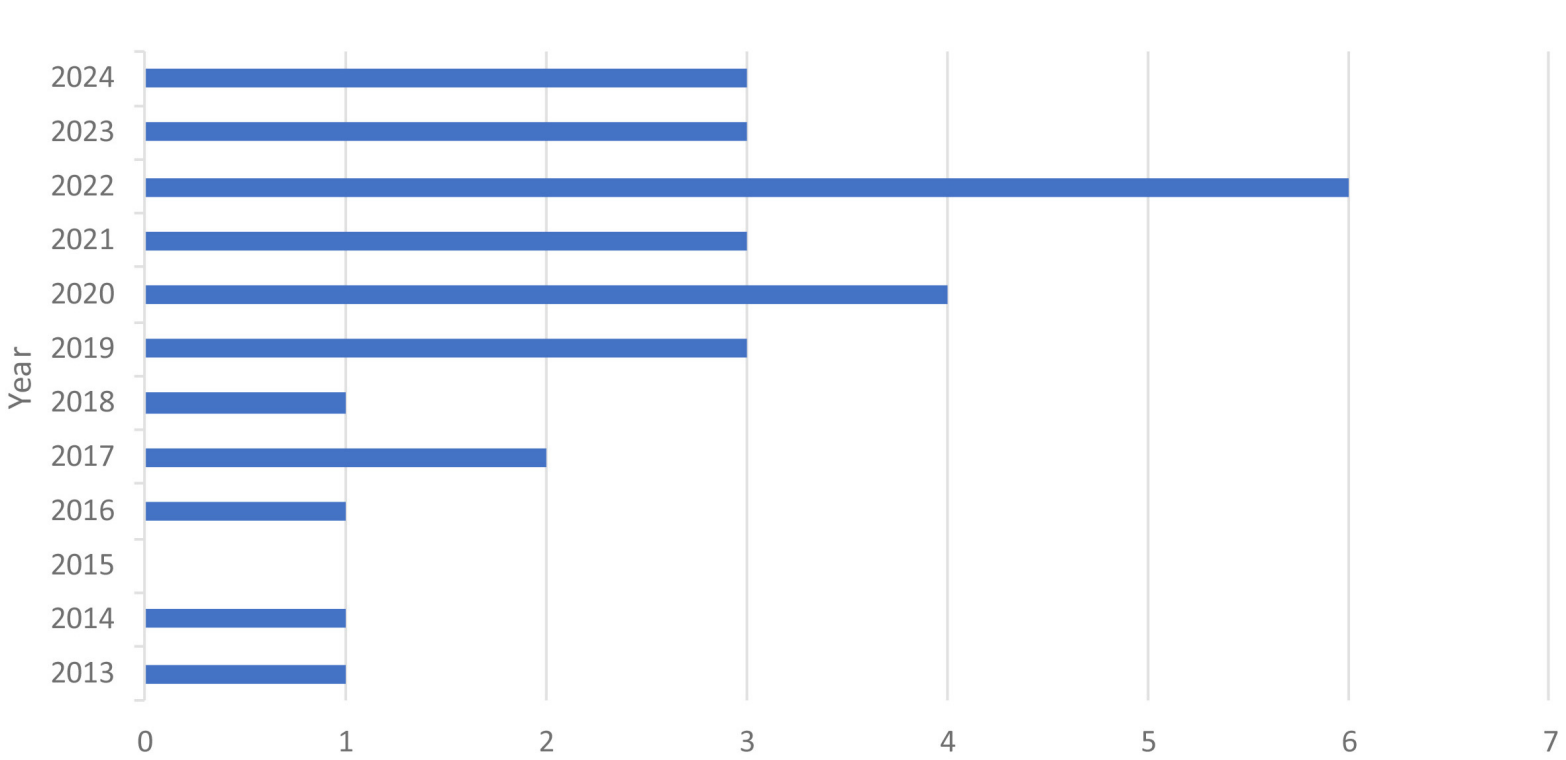
Temporal distribution of selected papers.

Temporal distribution of selected articles (Figure 3) dated from 2013–2017 are the early studies that establish core themes of occupational exposure and everyday practice. Swine workers in Romania expressed high concern but used little respiratory protection, pig farmers in Nepal faced widespread Japanese encephalitis exposure with inconsistent personal protection, Irish farmers reported raw milk consumption and limited well testing, Ugandan smallholder pig systems showed sanitation and housing constraints, and peri-urban producers in Ecuador normalized risk and accessed antimicrobials easily. These studies set the pattern of knowledge without consistent protection and highlight domestic and workplace routines as exposure pathways (1, 23, 31, 39, 41).

Between 2018 and 2020, the literature expanded from broad descriptions of occupational exposure toward more disease-specific evidence on zoonotic knowledge and practice. Studies from Cambodia, Uganda, Ethiopia, Bhutan, India, Vietnam, and Ghana drew attention to intention–behavior gaps, unsafe food and husbandry practices, and weak reporting or control along livestock and food-value chains (19, 21, 27, 34–36, 38). By 2021 until 2024, the field had become both larger and more analytically detailed, with newer studies from Ethiopia and Eritrea adding stronger worker-level evidence on brucellosis, bovine tuberculosis, risky dairy practices, and uneven zoonotic awareness, while research from Pakistan, Rwanda, Cameroon, Finland, Vietnam, Indonesia, and the United States increasingly linked behavior with organizational, ecological, and communication-related determinants (2, 16–18, 20, 22, 24–26, 28–30, 32, 33, 37, 40).

### 3.4 Risk perception and knowledge of zoonotic disease

Workers typically showed partial and uneven knowledge of zoonotic diseases, with marked differences between high profile infections and those that are less visible clinically or less widely discussed. In the brucellosis literature, dairy farmers in India showed very low awareness of brucellosis and limited recognition of its zoonotic implications (34). Similar patterns were reported in Eritrea and Central Oromia, Ethiopia, where many dairy owners and workers were unaware that contact with aborted fetuses, placentas, or unpasteurized milk could transmit infection, and often understood brucellosis more as a livestock productivity problem than as a direct occupational health threat (18, 20). Evidence from Ethiopian and Ugandan dairy and agro-pastoral settings further suggested that brucellosis was often understood more as a livestock productivity problem than as a direct occupational health threat, weakening perceived susceptibility and urgency for prevention (22, 26).

For parasitic and protozoal zoonoses, awareness was often even more limited. In Cambodian pig systems, a survey of porcine cysticercosis found that although farmers and traders were familiar with worms and meat quality issues, very few could name cysticercosis or explain its implications for human health, and risk perception remained low despite documented seroprevalence in pigs (36). A cross-sectional study of smallholder pig production in Uganda reported that many farmers did not perceive *Taenia solium* infection as a serious concern, normalizing practices such as free ranging pigs and open defecation that sustain the lifecycle of the parasite (23). Among small ruminant farmers in Cameroon, knowledge and perceived risk of toxoplasmosis and chlamydophilosis were generally low, with respondents often unaware of the specific zoonotic consequences of handling aborted materials or exposure of pregnant women to contaminated environments (29). Finnish dairy farmers showed somewhat better awareness of cryptosporidiosis as an emerging zoonosis, but still had gaps in understanding about asymptomatic infections and environmental persistence of the pathogen, indicating that even in high income contexts some relevant aspects of risk perception remain underdeveloped (40).

Vector-borne and viral zoonoses displayed a different but related pattern. Among pig farmers in Nepal, awareness that Japanese encephalitis exists and can affect people was relatively widespread, yet many farmers did not recognize that proximity of pig pens to human dwellings, stagnant water or lack of mosquito control increased local risk, which meant that their perceived susceptibility was often weaker than their general disease awareness would suggest (31). In Pakistan, livestock owners acknowledged the presence of ticks and their role in animal health problems, but a survey of tick-borne disease perception revealed that only a minority understood ticks as vectors of human infections or considered themselves to be at risk through handling infested animals (33). In Rwanda, livestock farmers recognized Rift Valley fever as an important disease and associated it with heavy rainfall and livestock morbidity, yet detailed knowledge of transmission routes, human symptoms and the role of vaccination was limited and varied by gender and previous exposure to outbreaks (25).

For zoonotic influenza and rabies, name recognition and perceived severity were generally higher. Swine workers in Romania and poultry farmworkers in Indonesia were well aware of avian and swine influenza viruses and often considered them serious threats both to animals and to human health, especially in the context of global media coverage of influenza pandemics (32, 39). Cattle owners in high-risk rabies areas in Bhutan almost universally recognized rabies as a fatal disease and were broadly familiar with the role of dog bites in transmission, which translated into a higher perceived need for vaccination of dogs and earlier seeking of post exposure prophylaxis compared to owners in low-risk areas (38). However, even in these cases, many workers struggled to link everyday occupational activities to risk and often underestimated the relevance of consistent preventive measures in apparently routine situations, for example handling pigs with mild respiratory signs or approaching free roaming dogs around cattle enclosures (32, 38, 39).

Broader assessments of health literacy and zoonotic knowledge confirmed that understanding of zoonotic risk is strongly conditioned by capacity to access and interpret information. In Vietnam, livestock farmers with higher health literacy scores were more likely to correctly identify zoonotic diseases and describe probable transmission pathways, whereas farmers with low literacy frequently relied on local heuristics that underplayed the role of environmental contamination and asymptomatic infection (35). General zoonotic knowledge among poultry farmers in Ghana and Cameroon and among Irish livestock farmers was also limited, with respondents often accepting that animals can make people sick but lacking precise understanding of which pathogens are involved, which tasks are most hazardous and what specific behaviors are protective (1, 27, 28). Overall, the review indicates that risk perception is fragmented and often poorly calibrated to actual exposure, particularly for infections that are endemic, environmentally mediated or lacking dramatic local narratives (Table 3).

**Table 3:** Study settings and type of zoonotic disease involved.

Research	Worker/Setting	Bacteria	Virus	Parasite	General
Suolaniemi et al. (40)	Dairy farmers			/	
Smith et al. (25)	Livestock farmers		/		
Rinchen et al. (38)	Cattle owners		/		
Rehman et al. (32)	Poultry farm & slaughterhouse workers		/		
Rabinowitz et al. (39)	Swine workers		/		
Pham-Thanh et al. (30)	Pig farmers & pigs		/		
Palomares Velosa et al. (2)	Dairy workers				/
Nankam Chimi et al. (29)	Small ruminant farmers	/			
Mahon et al. (1)	Farmers (esp. dairy)				/
Lowenstein et al. (41)	Peri-urban producers				/
Kungu et al. (23)	Smallholder pig farmers			/	
Kulabako et al. (22)	Cattle owners & workers	/			
Kemal et al. (19)	Cattle owners	/			
Hussain et al. (33)	Livestock keepers				/
Getahun et al. (18)	Residents incl. livestock owners	/			
Efrem et al. (20)	Dairy cattle owners & hired workers	/			
Dhakal et al. (31)	Pig farmers		/		
Deneke et al. (17)	Urban/peri-urban consumers	/			
Deka et al. (34)	Dairy farmers	/			
Cao Ba et al. (35)	Mixed livestock farmers				/
Bissong et al. (28)	Livestock handlers				/
Birhan (26)	Brucellosis synthesis	/			
Ayim-Akonor et al. (27)	Poultry farmers				/
Ayebare et al. (24)	Cattle keepers		/		
Atherstone et al. (21)	Pig traders/networks				/
Almaw et al. (16)	Cattle	/			
Adenuga et al. (36)	Pig farmers/traders			/	
Abukhattab et al. (37)	Broiler value chain				/

### 3.5 Preventive practices and the determinants of the perception-behavior gap

A major consistency observed in these studies was the difference between workers' self-assessed awareness of the zoonotic risks posed by pigs, as well as by animal handling in general and their normal activities in a production setting. Preventive measures such as pig confinement, latrine construction, safe carcass disposal, and avoidance of informal home slaughter were not widely implemented, even though some respondents indicated that it was possible for pigs to spread diseases to humans (23, 36). The lack of money, sufficient housing space, deeply ingrained cultural practices and the lack of regulations in place prevented many from making safer adjustments to their daily operations.

Similar findings were observed among dairy production systems. Several studies conducted in rural communities of India reported the frequent consumption of raw or under-cooked milk, contact with reproductive materials, including placentas and aborted fetuses without protective measures and less use of personal protective equipment (17, 18, 20, 34), even when aware of potential disease risks to humans. Dairy farmers in Ethiopia reported similar risky behaviors such as handling raw milk from infected animals and inadequate milking procedures, which they attributed to the prevailing culture and their affinity for dairy products. The study highlights the link between behavioral changes required for control and several other factors including food preferences, perceived practicality of safe procedures in their workflow, habit, as well as differing perceptions of perceived hazard of animal products between men and women in the rural setting.

PPE use and biosecurity practices varied considerably among poultry farmers and livestock value-chain workers. Poultry farmers recognized risks from zoonotic diseases in Ghana and Cameroon but said they rarely used disposable gloves, masks, safety coveralls and cleaning agents, primarily because they were prohibitively expensive, uncomfortable to wear and breathe through due to heat or they did not worry about enforcement daily (27, 28). Livestock workers knew the seriousness of the Crimean Congo hemorrhagic fever but few wore the specified precautions during slaughter and when handling cattle due to there was still not enough in the way of resource access to support it and also because the severity of the disease was often overlooked (24).

Factors for these behaviors were found at individual, social, environmental and organizational levels. Education, as reflected by improved health literacy, led to better knowledge of zoonotic transmission routes in Vietnam, Cameroon and Ireland, but knowledge alone did not lead to safer behavior (1, 28, 29, 35). Moreover, gender and social roles affected both risk and agency among individuals. For example, men and women differed in Rwanda about their knowledge of Rift Valley fever transmission, the types of methods used for mitigating Rift Valley fever and preferred communication sources for disease information and livestock were integrated into family activities in Ecuador and close interaction was taken for granted among family members in home settings (25, 41).

At the organizational level, the translation of knowledge into action depended strongly on workplace support and enabling conditions. Dairy workers on Colorado farms that enjoyed positive peer and supervisor morale, open communication and a positive safety culture generally demonstrated lower occupational exposure than did those with negative workplace attitudes (2). Romanian and Indonesian livestock farmworkers were aware that influenza could be transmitted to humans, but personal use of masks, gloves or other protective equipment was not routine unless supplies were readily available, commonly used and integrated into workplace operations (32, 39). Private wells in Ireland, for instance, seldom were tested because homeowners were unaware of the issue or lacked access to laboratory services while in Uganda, poor infrastructure of pig management systems contributed to disease transmission, with common lack of sanitation facilities, inadequate handwashing provisions, irregular deworming and shared resources and spaces (1, 23).

Taken together, these findings indicate that protective behavior was shaped by several interacting determinants. Knowledge and experience of disease mattered, but only as mediated by, among other factors, gender-based labor divisions, cash flow restrictions, the structure of employment and workloads, availability of animal health services, the presence of water supply and sewage systems or the delegation of animal management. Protection practices are consistently used where work is adequately organized with supervisory staff or the organization provides resources and in the presence of supportive conditions, conversely, protection use is poorest where individual workers meet the cost of protective clothing, washing facilities, disease prevention and therapy out of their own pocket, with poor facilities, minimal support from extension staff or where several actors share responsibility.

## 4 Discussion

### 4.1 Key knowledge gaps and behaviors influencing zoonotic disease risks

This review shows that workers at the human–animal–environment interface commonly carry out high-risk activities while holding only partial or inaccurate understandings of zoonotic disease risks. Across the different diseases and production systems, a recurrent pattern is that workers know that animals can transmit disease in a general sense but have limited knowledge of specific pathogens, transmission routes and prevention strategies. Evidence from Ethiopian dairy settings illustrates a broader pattern across the review: workers often understood zoonotic disease through the lens of animal productivity and routine husbandry rather than through an occupational health framework, which weakened perceived personal susceptibility and urgency for prevention (18, 20, 34). Similar patterns were also observed in India and Eritrea, where workers were routinely exposed to brucellosis-related hazards but often lacked disease-specific understanding of how those exposures affected human health (22).

For parasitic zoonoses, knowledge gaps were even more noticeable. Pig farmers and traders in Cambodia and Uganda frequently normalized practices such as free roaming pigs, open defecation and informal home slaughter, while awareness of porcine cysticercosis and *Taenia solium* as zoonotic threats remained low (23, 36). Small ruminant farmers in Cameroon and dairy farmers in Finland were often unfamiliar with the zoonotic implications of toxoplasmosis, chlamydophilosis and cryptosporidiosis, particularly for pregnant women and children, which resulted in a relatively low perception of personal or household risk despite occupational exposure (29, 40).

High profile vector-borne and viral zoonoses showed slightly better name recognition but still revealed important knowledge gaps. Pig farmers in Nepal and livestock owners in Pakistan were aware of Japanese encephalitis and tick-borne diseases in broad terms but did not consistently associate mosquito habitats, pig housing location or tick burden with heightened human risk (31, 33). Livestock farmers in Rwanda and cattle owners in Bhutan recognized Rift Valley fever and rabies as severe diseases, yet detailed understanding of human symptoms, incubation periods and optimal mitigation strategies remained incomplete, with notable differences between groups depending on gender and risk zone (25, 38). Swine workers and poultry farmworkers in Europe and Indonesia were familiar with zoonotic influenza but often lacked clarity about when and how specific tasks such as handling mildly ill animals or cleaning poultry houses substantially increased infection risk (32, 39).

These knowledge gaps have direct behavioral consequences. The review documents a wide range of unsafe but routine practices, such as consumption of raw or inadequately boiled milk, handling of animal birth products without protection, poor carcass and offal disposal, lack of latrines and environmental contamination, and irregular or absent use of gloves, masks and boots during high-risk tasks (17, 20, 27, 28, 34). Many workers described these behaviors as normal features of daily life, often passed down through generations or embedded in local food cultures. Even where knowledge was relatively good, as in some influenza, rabies or cryptosporidiosis contexts, behavior lagged behind, which underscores that risk related behavior is not simply a mechanical function of awareness (32, 38, 40).

### 4.2 The role of socio-cultural, economic and environmental context in shaping risk perception

Risk perception across the reviewed studies was not simply an individual cognitive judgment; it was shaped by the material, social, and organizational conditions under which people worked. Education and health literacy improved recognition of zoonotic transmission routes in several settings, including livestock farmers in Vietnam, where higher health literacy was associated with better understanding of zoonotic disease pathways, and farmers in Ireland, where veterinarians were the most commonly cited information source for farm disease risks (1, 35). Yet better knowledge did not reliably produce safer practice. In Ghana, poultry workers identified personal protective equipment (PPE) as important but rarely used gloves, masks, or overalls in daily farm work, despite regular handwashing and substantial work experience (27). Similarly, in Romania, swine workers reported high concern about zoonotic influenza but very low uptake of respiratory protection and seasonal vaccination, showing that concern and awareness alone did not guarantee institutionalized prevention (39).

Social roles and patterns of information flow also shaped exposure and agency. In Rwanda, male and female livestock farmers differed in RVF knowledge, mitigation strategies, and communication channels, while female farmers were less likely to be regarded as credible sources of advice, suggesting that information access and authority were distributed unevenly within the same occupational setting (25). In Nepal, pig farmers commonly relied on veterinarians, peers, and media for information about Japanese encephalitis, while prevention practices remained uneven and vaccine uptake was almost absent, indicating that social networks influenced awareness but did not necessarily overcome structural barriers to prevention (31). In Ecuador, livestock keeping was embedded in household life, intergenerational practice, and economic necessity, with close human–animal contact normalized as part of everyday survival rather than interpreted primarily as a preventable health hazard (41).

The strongest cross-study pattern was that organizational and infrastructural conditions mediated whether awareness could be translated into action. In Colorado dairy farms (2), researcher found that supportive supervisors and coworkers, strong communication, and a positive organizational environment were associated with lower zoonotic exposure, while negative workplace perceptions increased risk. The same study argued that organizational factors are relevant drivers of preventive behavior and that instruction on face and respiratory protection should be strengthened where exposure is plausible. In Uganda (23), study showed that safer conditions were linked to protected water sources, access to latrines, and handwashing infrastructure; pigs from households using protected water or having usable latrines were less likely to have cysticercosis, while only about half of farmers had clean water near latrines and fewer still used soap. In Ireland, private-well testing was infrequent despite the health relevance of potable water, again indicating that environmental health protection depends partly on infrastructure and compliance systems rather than only on awareness (1). In Palestine (37), it is found that workers often reported good hygiene awareness, yet observational findings pointed to poor hygiene practices, insufficient infrastructure, weak veterinary and laboratory capacity, fragmented authority, and inconsistent application of law. Taken together, these findings suggest that workers do not simply fail to act because they underestimate risk; rather, risk becomes normalized when safe behavior is not adequately supported by employers, infrastructure, or institutions.

### 4.3 Policies and interventions: what is known and what is needed

A central implication of the reviewed evidence is that interventions focused only on awareness-raising are unlikely to be sufficient. Across multiple studies, workers recognized zoonotic risks in broad terms but did not consistently adopt protective practices where PPE was unavailable, uncomfortable, weakly enforced, or disconnected from routine work processes. In Romania, workers were aware of influenza guidance and many reported concern about infection, yet respirator use remained rare and vaccination uptake remained very low; the authors noted that low availability of respirators, limited training, discomfort, and weak farm-specific standards could all contribute to this gap (39). In Ghana, poultry workers washed hands regularly but still reported almost no mask or glove use and worked in farms where footbaths were often absent and waste was commonly left on site, indicating that hygiene messages were not matched by enabling farm conditions (27).

The evidence therefore points to the need to shift part of the responsibility for prevention upward, from individual workers to employers, managers, veterinary systems, and regulators. Study showed that zoonotic exposure was shaped not only by worker knowledge but also by safety climate, supervisory communication, and whether farms meaningfully included zoonotic disease prevention in their training and information systems (2). In Palestine (37), it is identified scarcity of public slaughterhouses, insufficient coordination between authorities, poor communication between public and private sectors, insufficient monitoring, and inconsistent application of the law as major obstacles to safer food production. In Ireland (1), researcher highlighted the continued risks posed by infrequent well testing and unpasteurized milk consumption despite longstanding hygiene guidance. In Uganda, researcher showed that sanitation and water access were part of the transmission environment itself, not merely background conditions (23). These studies collectively suggest that effective prevention depends on whether institutions provide safe infrastructure, practical training, environmental hygiene support, and enforceable standards that make safer behavior feasible in everyday work.

Accordingly, the most credible policy direction emerging from this review is a multi-level One Health approach that combines education with structural provision. The reviewed studies support interventions such as employer-funded and routinely enforced PPE, safer work organization, better supervision and communication, access to clean water and sanitation, strengthened veterinary and laboratory support, improved slaughter and waste-management infrastructure, and communication strategies tailored to local social networks and gendered information pathways. This is consistent with evidence from Rwanda and Nepal, where communication effectiveness depended on who was trusted and how information circulated, and with findings from Ecuador and Palestine showing that broader livelihood and governance conditions shape whether risk reduction is practical or merely aspirational (25, 31, 37, 41). Overall, the literature suggests that durable change will come not from telling workers to be careful in isolation, but from reorganizing the environmental and institutional conditions under which they work.

## 5 Conclusion

Workers of livestock farms and mixed farming system generally underestimate zoonotic disease risk, are ignorant to key transmission routes of zoonoses, inability of workers to integrate risk-awareness in their daily works with current work practices and, misunderstanding of personal hygiene importance for prevention of infection of zoonoses. These patterns were shaped by the interaction of partial knowledge, socio-cultural norms, livelihood pressures, working and housing conditions, and environmental exposure.

The existing evidence base provides a strong foundation for building understanding on zoonotic disease risk perceptions and preventive practice within the agricultural occupational groups of farm and livestock systems. However, there is less empirical evidence on these from plantation occupation settings. These three review questions have close correlation. Misperceptions of risk and risky practices occur because knowledge on zoonotic diseases is not complete or the knowledge provided to the farm workers is not equitably distributed and poorly interpreted as practical guidelines for their daily work. Risk perception and practice is influenced by socio-economic and environmental contexts, cultural aspects, workplace settings and so on, hence workers may perceive less risky and even norm their exposure to zoonotic pathogens.

Accordingly, knowledge awareness-raising alone will probably lead nowhere. Therefore, more efficient interventions will involve multi-level approach and are context specific using One Health perspective. The interventions may combine context-specific communication along with improvements of infrastructure, sanitation, workplace organization, employer responsibility and supported by legal compliance. Prevention and control of zoonoses should be integrated in occupational health and environmental management of livestock and farming systems. On the other hand, the current evidence base is not fully comprehensive and future research needs to be focused on the other important sectors such as plantation labor where varied employment forms, housing, environmental exposure and managerial systems may play diverse roles in determining zoonotic disease risk to labor.

## Data Availability

The original contributions presented in the study are included in the article/Supplementary material, further inquiries can be directed to the corresponding author/s.
